# Enhanced Anti-Ultraviolet and Thermal Stability of a Pesticide via Modification of a Volatile Organic Compound (VOC)-Free Vinyl-Silsesquioxane in Desert Areas

**DOI:** 10.3390/polym8080282

**Published:** 2016-08-04

**Authors:** Derong Lin, Maozhu Kong, Liangyu Li, Xindan Li, Xingwen Zhang

**Affiliations:** 1 College of Food Science, Sichuan Agricultural University, Ya’an 625014, China; 18227589574@163.com (M.K.); 18227584828@163.com (L.L.); 18227589580@163.com (X.L.); 2 State Key Laboratory of Urban Water Resource and Environment, Harbin Institute of Technology, Harbin 150090, China; zhangxinwen@hit.edu.cn

**Keywords:** silsesquioxane (SSO), pesticide (citral), polymerization, polymer delivery system, repellency

## Abstract

Due to the effect of severe environmental conditions, such as intense heat, blowing sand, and ultraviolet light, conventional pesticide applications have repeatedly failed to adequately control mosquito and sandfly populations in desert areas. In this study, a vinyl silsesquioxane (VS) was added to a pesticide (citral) to enhance residual, thermal and anti-ultraviolet properties via three double-bond reactions in the presence of an initiator: (1) the connection of VS and citral, (2) a radical self-polymerization of VS and (3) a radical self-polymerization of citral. VS-citral, the expected and main product of the copolymerization of VS and citral, was characterized using standard spectrum techniques. The molecular consequences of the free radical polymerization were analyzed by MALDITOF spectrometry. Anti-ultraviolet and thermal stability properties of the VS-citral system were tested using scanning spectrophotometry (SSP) and thermogravimetric analysis (TGA). The repellency of VS-citral decreased over time, from 97.63% at 0 h to 72.98% at 1 h and 60.0% at 2 h, as did the repellency of citral, from 89.56% at 0 h to 62.73% at 1 h and 50.95% at 2 h.

## 1. Introduction

Mosquitoes are important vectors of arthropod-borne viruses such as yellow, dengue and chikungunya fevers, causing worldwide outbreaks of severe epidemic diseases every year [1]. Researchers have been studying the prevention of mosquito bites for centuries, and they have been using pesticides to kill mosquitoes [2]. However, most pesticide consists of organic substances which are volatile or unstable in desert areas [3]. The other problem is that pesticides mixed in sun protection cream (SPC) are non-resistant to ultraviolet radiation, which can cause skin damage including wrinkles, lowered immunity against infection, aging skin disorders, sunburns and cancer [4,5]. Naturally occurring terpene oils have been found to be less hazardous substitutes for synthetic pesticides. One common constituent of these oils is citral, which has shown promise as both insect repellent and insecticide [6,7,8,9,10].

In recent years, the growing interest in polyhedral oligomeric silsesquioxanes (POSS) or silsesquioxanes (SSO) is evidenced by the increased number of studies published and from both experimental and theoretical investigations due to the unique physical and chemical properties of silsesquioxanes [11,12,13,14,15,16,17,18]. One of the important advantages of these SSO nanostructured hybrids is that they are too large to have appreciable vapor pressures and hence are free of volatile organic content (VOC) [19,20]. Another important advantage of SSO hybrids is a high sun protection factor (SPF) for protection from UV radiation and the prevention of skin damage [21,22]. As mentioned above, SSO is a good candidate as a chemical ingredient for modifying pesticide creams to enhance their stability and prevent UV radiation skin damage in desert areas. In this study, the first aim is to connect a pesticide (citral) to a vinyl-SSO (VS) via emulsion polymerization to determine if the VS prevent the pesticide from volatilizing or moving while decreasing the absorbed UV radiation.

Due to the absence of VOC in SSO materials, these systems possess inherently low flammability and thus are not subject to uncontrolled polymerization [23]. There are three polymerizations in the blend of the citral and VS system: two radical self-polymerizations of both citral and VS systems and one copolymerization for the connection of the citral and VS.

Vinylsilsesquioxanes [24,25] and even VMS [26] are known to react under free radical conditions. While generally monomers such as VMS are better copolymerized with more reactive organic vinyl monomers [27], the polyvinylsilsesquioxanes have sufficient vinyl groups to permit the oligomeric materials to be covalently linked together and even crosslinked. We originally had been interested in whether or not the citral would react under free radical conditions to covalently link the citral with the VS. We used mass spectrometry to structurally characterize the VS-citral and UV-Visible spectroscopy to evaluate the transparency of the materials. Thermal gravimetric analyses were used to evaluate how much citral was in the VS-citral material.

## 2. Experimental Section

### 2.1. Materials

Commercial vinyltrimethoxysilane (VMS, Sigmas Aldrich, St. Louis, MO, USA, at a purity of 96.0%–97.0%) was used as a precursor in the study of hydrolytic condensation to prepare vinyl-SSO, ethanoland tetrahydrofuran (THF) were used as solvents (99.7% purity) and formic acid was employed as a catalyst (88% purity); citral (Alfa Aesar, Ward Hill, MA, USA, 95%) can be selected as a main component of the modified pesticide; sodium dodecyl sulfate (SDS) as a surfactant; and potassium persulfate (PP) as an initiator were supported by Tianjin BoDi Chemicals and Tianjin Fine Chemicals (Tianjin, China), respectively. All of the above reagents are of analytical purity, used without further treatment.

### 2.2. Synthesis of Vinyl-SSO

For the hydrolytic condensation reaction, VMS was placed in beakers. Ethanol was added to give a 3:1 molar ratio with respect to Si. The polycondensation was carried out in the presence of formic acid, added in a 0.5:1 molar ratio with respect to Si. The corresponding molar ratio of water to Si was 1:1. The beaker was sealed with a plastic film and left for a day at 35 °C. Then needle-sized holes were made in the plastic film and the reaction was continued for another day at the same temperature. Finally, the plastic film was removed and the reaction continued for one more day at 65 °C. The reaction products were homogeneous viscous liquids that could be completely dissolved in different solvents such as tetrahydrofuran (THF), chloroform and ethanol. The SSO based on VMS will be denoted as VS (VMS-SSO).

### 2.3. Free Radical Polymerization of VS in Citral Oil in Water Emulsion

**Scheme 1:**
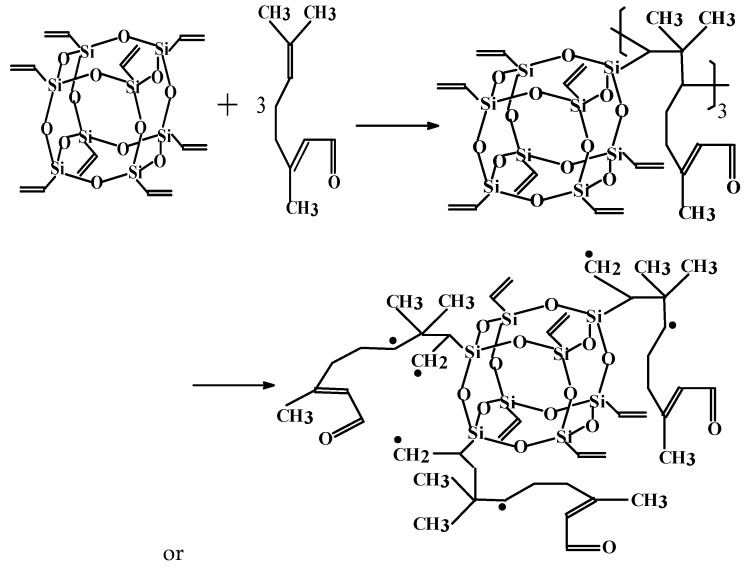
The copolymerization (1) for the connection of VS and 3 citrals, indicating that the reaction of the VS and citral favors their connection in the blend of VS, citral and a suitable initiator.

The emulsion polymerization was employed to prepare the compound of the pesticide modified with VS as shown in Scheme 1. Firstly, VS (1 g) and citral (8 g) were added into a 250 mL two-neck round bottom flask with 140 mL of distilled water and stirred continuously for 4 h, and then (1) 4.00 g SDS and 0.05 g SSP were added into the flask at 80 °C for 1 h; (2) 0.08 g PP and 5% NaOH solution were added dropwise into the solution and stirred at 80 °C. After that (1) the solution was stirred continuously for additional 12 h under 40 °C; (2) a filtration of reaction residues had to be performed; (3) THF solution was dropped into a beaker of methanol and rapidly stirred. Finally, the resulting product was collected and dried in a vacuum oven for 24 h. The pesticide modified with VS is denoted to VS-citral.

### 2.4. Characterization and Measurements

The structures of the VS, citral and VS-citral were characterized using Fourier transform infrared spectroscopy (FTIR, Avatar 360 device, Nicolet, Thermo Fisher Scientific, Waltham, MA, USA) and Biflex III matrix-assisted ultraviolet laser desorption/ionization time-of-flight mass spectrometry (UV-MALDI-TOF MS, Bruker, Billerica, MA, USA), and the equipment measuring conditions of UV-MALDI-TOF MS for characterization of the VP-pesticides in detail were similar to that in the previous work [28,29].

For the properties, a UV-3101PC Scanning Spectrophotometer (SSP, Shimadzu Corporation, Kyoto, Japan) device was used to measure the film transparency in a range of wavelengths between 280 and 400 nm (UV-A and UV-B region). TGA was performed on TA Instruments ZRY-2P equipment (Shanghai Balance Automotive Equipment Co., Shanghai, China) under nitrogen atmosphere at a heating rate of 20 °C·min^−1^ from 0 to 800 °C.

### 2.5. Mosquito Repellent Bioassays

*Aedes albopictus* (Skuse, Beijing, China) mosquitoes were used for repellent testing. Mosquito larvae were obtained from the Department of Entomology of Chinese Center for Disease Control and Prevention (China CDC, Beijing, China). The larvae were reared at 28 °C and 60% relative humidity. Adult mosquitoes were fed and maintained on a 10% sucrose solution. Two kinds of mosquito repellents, 5% citral (Alfa Aesar, Ward Hill, MA, USA, 95%), and 5% VS-citral, were purchased and prepared. Aliquots of 1.5 mL were applied to volunteers’ forearms to test repellent efficacy [30]. A test cage (50 × 60 × 50 cm^3^) was constructed with a metal frame to make decontamination easier. A fabric sleeve was added to the front side of the test cage to allow access by a human forearm. A patch containing repellent agent was applied to clean skin on the volunteer’s forearm and allowed to remain on the skin for 36 h. Volunteers were not permitted to remove or wet the patch during this time [31]. After 36 h, the patch was removed, and initial results were determined. The patch was marked region of the forearm and results were determined 72 h after initial patch placement. A modified version of the World Health Organization Pesticide Evaluation Scheme (WHOPES) arm-in-cage assay was used [32]. Three hundred female mosquitoes (age 6 days), which had never received a blood meal, were placed into each test cage and starved of their sugar diet for 12  h before the test. A 1.5 mL aliquot of each repellent solution was applied evenly on the right forearm between the wrist and elbow using a pipette and allowed to dry for approximately 5 min. The untreated left arm was placed into a test cage for 3 min and the number of mosquitoes landing on that arm was counted. Repellent-treated right arms were placed into the test cage for 3  min at 1  h intervals, VS-citral-treated arms for 2  h, and arms treated with citral for 2  h. The number of mosquitoes that landed on or bit that arm was recorded every hour.

Repellency (*R*) was calculated using the formula [33].

(1) $$R(\%)=(1-\frac{T}{C})\times 100\%$$

where *C* is the number of mosquito bites on the control arm and *T* the number of bites on the treated arm.

### 2.6. Statistical Analysis

All experiments were performed in triplicates. The repellency of the control and treated arms was compared using *F*-tests, with a *P* value 0.05 considered statistically significant. A commercial software program (SPSS 18.0, SPSS Inc., Chicago, IL, USA) was used for statistical analysis.

## 3. Results and Discussion

### 3.1. Determination and Validation of the Main Reaction

**Scheme 2:**
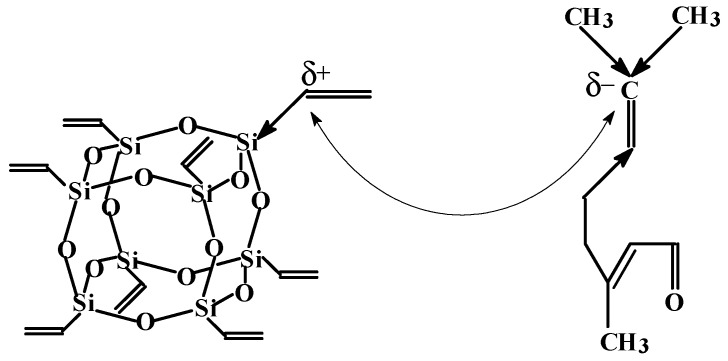
The polarity effects on the double bonds of both VS and citral for the connection: Si is an electron-attracting atom and C is an electro-donating atom, which two C atoms in the VS and citral possess positive and negative charges, respectively.

For the blend of VS and citral, there are three co-polymerizations or oligomerizations: (1) one copolymerization for the connection of VS and citral (see Scheme 1), (2) a radical self-polymerization of VS and (3) a radical self-polymerization of citral. Theoretically, the expected copolymerization (1) is easy due to the polarity effects on the double bonds of both VS and citral for the connection (see Scheme 2). In contrast, self-polymerizations (2) and (3) are difficult and need a suitable initiator [34].

The oil in water emulsion polymerization involved the free radical reaction addition to the vinyl groups of the VS. The citral and VS formed the oil phase dispersed in a continuous water phase with the water-soluble, persulfate free radical initiator. The SDS was used as a surfactant to stabilize the micelles in the emulsion. Because of the large number of vinyl groups on every VS oligomer, it is theoretically possible for the product of the emulsion polymerization to be completely insoluble. However, due to the lower reactivity of these vinyl groups attached to silicons [24,26,27], chain polymerization that would lead to highly crosslinked networks was less likely than free radical chain transfer that would allow interconnection of oligomers with much less crosslinking. This was borne out by the small amount of insolubles, presumably crosslinked VS, filtered off during the isolation of the soluble VS-citral. This was discounted as a serious possibility by the known propensity of citral and related terpenes to undergo free radical cyclization [35,36,37] instead of polymerization and with our mass spectrometric and thermal gravimetric analyses below.

### 3.2. Characterization of FTIR Spectrum 

**Figure 1:**
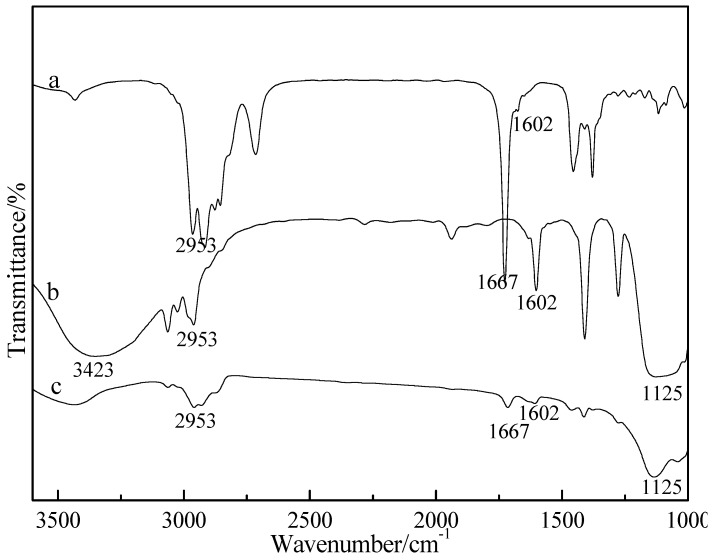
FTIR spectra of (**a**) citral, (**b**) VS and (**c**) VS-citral.

Figure 1a shows the FTIR spectrum of the citral where typical bands at 1667, 2953 and 1602 cm^−1^ are primarily ascribed to the stretching of C=O, H–C and C=C groups, respectively. The FTIR spectrum of VS is shown in Figure 1b; The typical band at 3423 cm^−1^ is primarily ascribed to the stretching of the OH of Si–OH groups that are hydrogen bonded possibly to other Si–OH groups and from water hydrogen bonded to itself and to the Si–OH groups. The obvious bands at about 1120–1129 cm^−1^ derive from Si–O–Si antisymmetric stretches, and 1602 cm^−1^ is ascribed to the stretching of the C=C group. A relatively small band of C-H groups at 2953 cm^−1^ was also visible [28]. Figure 1c shows the FTIR spectrum of the VS-citral where all typical bands in both the citral and VS are still obviously visible except that the band at 1602 cm^−1^ ascribed to the stretching of the C=C group decreased, indicating that polymerizations of double-bond groups occurred.

### 3.3. Characterization of Mass Spectrometry

**Figure 2:**
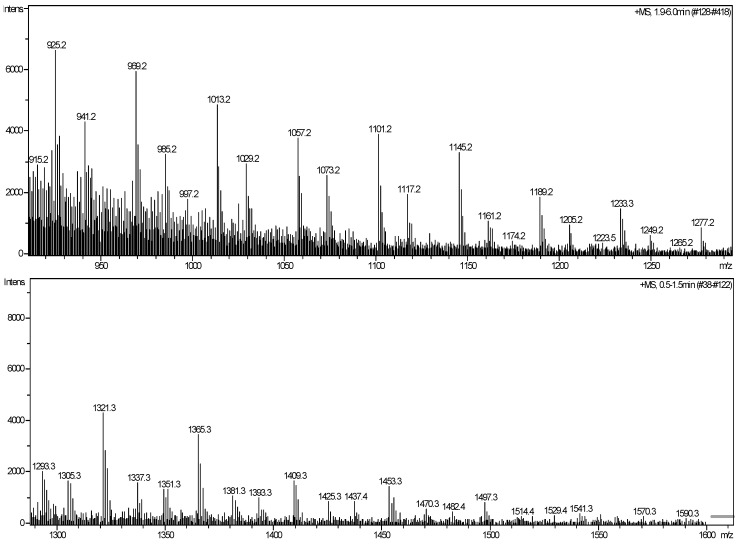
MALDI-TOF mass spectra in the *m*/*z* = 925.2–1590.3 Da range correspond to the VS-citral oligomers.

Figure 2 shows MALDI-TOF mass spectra in the *m*/*z* = 925.2–1590.3 Da range correspond to the VS-citra oligomers, and the proposed structural formulas corresponding to the peak data were assigned with the generic formula (RSiO_1.5_)*_n_* (*n* = even number ≥ 6; complete cage structure) or [RSiO_1.5 – (*x* + *y*)/2*n*_]*_n_*(OH)*_x_*(OCH_3_)*_y_* (*n* = 4, 5, 6...; *x*, *y* = 1, 2, 3...; incomplete cage, ladder and branched linear structures) (as seen in Table 1) [38]. These peak data exhibit excellent compliance between the experimental measurement value (*m*/*z*) and the calculated molecular weight according to ion adducts (M + H^+^ or M + Na^+^ or M + K^+^) [28,39,40].

**Scheme 3:**
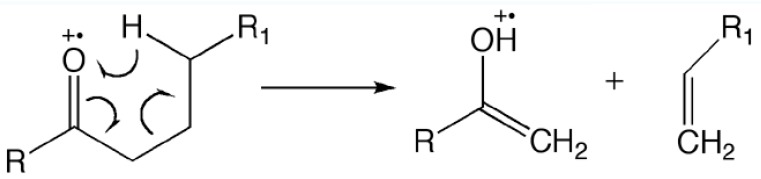
One supposed sample of McLafferty Rearrangement (mass spectrometry), deleting an enolic fragment (mass: 44) and leaving the rest of the parts in the VS-citral.

There are individual larger frames containing *T*_n_ clusters in Figure 2. Except for T_5_ and T_6_ clusters, each larger frame contains two main peaks. One part of the peak data in the small square frames for T_7_–T_13_ clusters (see Figure 2) is the VS-citral masses after the McLafferty Rearrangement (mass spectrometry) of one citral where an enolic fragment (mass: 44) is deleted, and the balance of the peak remains (see Scheme 3); these peaks are called rearrangement peaks [41,42]. The other main peaks express the *T*_n_ species of which each SSO connects only three citrals to stabilize pesticides. Beyond three connected, the citrals will be rearranged as the fourth citral is difficult to connect due to a steric effect.

### 3.4. Transparency in the UV-B Region

**Figure 3:**
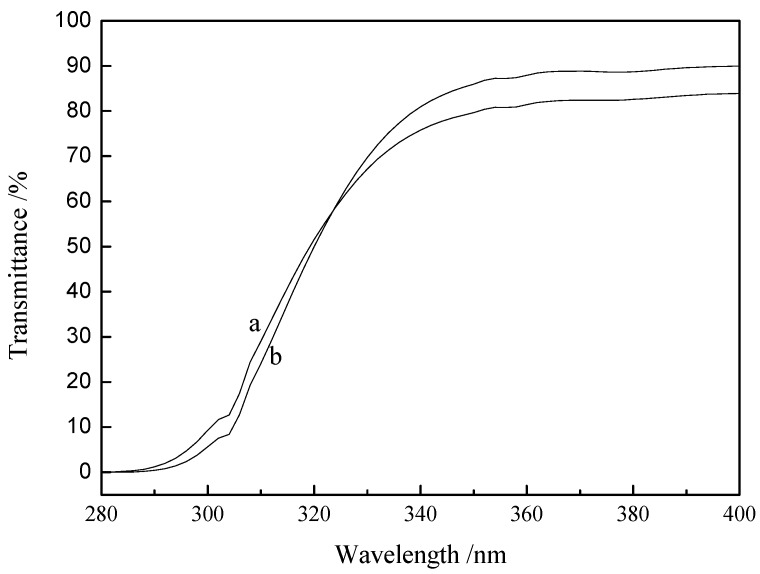
The transparency of citral (**a**) and VS-citral (**b**) measured in the range of the UV-A and UV-B region (280–400 nm).

Measured in the range of the ultraviolet-B (UV-B) and ultraviolet-A (UV-A) region (280–400 nm) (see Figure 3), the average transparency value of the VS-citral oligomer in the range of the UV-B spectrum (280–320 nm) is only about 13%, indicating this hybrid has a better light absorption in the UV-B spectrum range and can provide a physical barrier for blocking the UV-B radiation absorbed by the skin, highlighting the functionality of the VS material as sun protection factor (SPF) ingredients in the anti-mosquito cream to prevent UV radiation skin damage in desert areas.

### 3.5. Thermogravimetric Analysis of VS-Citral

**Figure 4:**
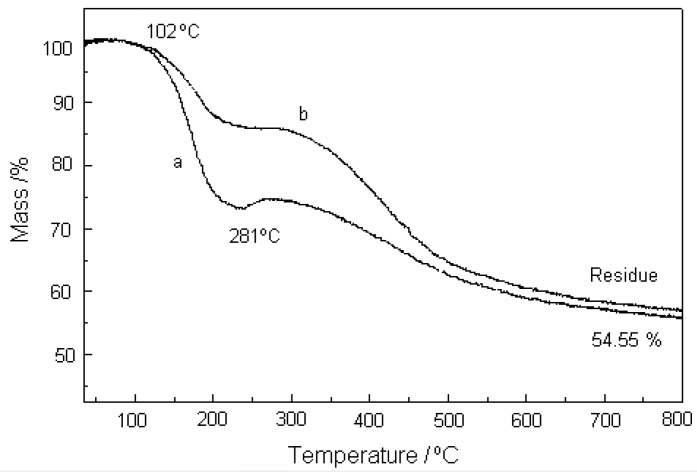
The thermal gravimetric (TG) curves of citral (**a**) and VS-citral (**b**) under the conditions of an increased speed of 20 °C/min and a temperature range of 0–800 °C.

Thermogravimetric analysis shows that there is about 15% citral released from VS-citral. Figure 4 shows the thermal gravimetric (TG) curves of citral (a) and VS-citral (b). There are two main steps of weight loss on the TG curve. The first weight loss in both samples is the step at 102 °C. We believe this is due to the evaporation of citral (bp 219 °C). Citral is known to react with oxygen and increase in mass above 200 °C is likely due to the formation of peroxide species and their reaction to form polymeric species. The increase is not seen in the VS-citral because the polysilsesquioxane isolates the molecules of citral from one another, reducing the rate of reactions. The second step starting from 281 °C to the end mainly taking place at 700 °C corresponds to the degradation of the samples, especially for the decomposition of the organic corner groups. Char residue obtained from the TG curve is a parameter reflecting the thermal stability of the samples. The char residues are taken as the weight percentage of the sample remaining after the TG test which is 54.55% for the citral and slightly lower for the VS-citral due to the content of inorganic groups in the structure. Observed from the TG test (especially in the second weight loss step as shown in Figure 4), VS-citral possesses superior thermal stability compared to the citral. Owing to the SSO peculiar nanometer structure, it shows great potential as a modifier to improve the properties of pesticides, especially for thermal properties. The mass of citral char left behind depends on the rate of heating (and evaporation) of the citral and the concentration of oxygen in the gas flowing over the sample. The amount of char residue for the VS is, at its theoretical lowest, the mass of silica obtained from complete combustion of the organic constituent or 38%. The greater char residue is consistent with the formation of silicon oxycarbide materials from VS materials. Thermolysis of polyvinylsilsesquioxanes typically has char yields between 70%–90%, indicating that the actual loading of citral available in the VS-citral system may be higher than 15%.

### 3.6. Repellent Effect for Citral and VS-Citral.

Citral and VS-citral were tested on eight volunteers each. Botanical mosquito repellents such as citral and VS-citral according to the China Food and Drug Administration (CFDA) guideline. Table 2 shows the mean numbers of mosquitoes landing on untreated (control) and treated forearms of volunteers over 3 min. The mean number landing on the untreated forearms of 8 volunteers over 3 min was 38.38 ± 2.96. Testing of the repellency of treated forearms every hour for 2 h showed perfect repellency for 5% VS-citral over the first 2 h. Three (V3, V5, and V8), eight (V1, V2, V3, V4, V5, V6, V7 and V8), and eight (V1, V2, V3, V4, V5, V6, V7 and V8) volunteers were bitten at 0, 1, and 2 h, respectively, making the repellency at these times 97.63% ± 0.72%, 72.98% ± 3.11%, and 60% ± 4.23%, respectively. These results indicated that 5% VS-citral had >60% repellency for 2 h.

Repellency tests of 5% citral were performed on 8 volunteers. A mean (±SE) of 23.38 ± 0.66 mosquitoes landed on their untreated left forearms during exposure to 300 mosquitoes for 3 min (Table 3). Repellency tests of 5% citral were performed at application and 1 h and 2 h later. Repellency at 0, 1, and 2 h was 89.56% ± 3.02%, 62.73% ± 5.31%, and 50.95% ± 6.10%, respectively. These results indicated that 5% citral had >50% repellency for 2 h.

## 4. Conclusions

A pesticide (citral) can be modified with VS to enhance anti-ultraviolet and thermal stability in desert areas. During the modification, there were three double-bond reactions in the blend of citral and VS in the presence of an initiator. The product of the main reaction (VS-citral) was characterized by FTIR and MS spectra. From the MS spectrum, there were two *T*_n_ sub-clusters in the main *T*_n_ cluster: one *T*_n_ sub-cluster indicated a normal *T*_n_ cluster with three citrals and the other sub-cluster indicated that one citral among three in the normal *T*_n_ cluster deleted an enolic fragment (mass: 44) after the McLafferty Rearrangement.

The measured anti-ultraviolet and thermal stability of the VS-citral oligomer gives it great potential as a modifier to improve the properties of pesticides due to the SSO peculiar nanometer structure.

We have successfully prepared a silsesquioxane-based delivery system for citral that contains more than 15% citral for slow release to provide long-term protection against insect pests. The material is easy to prepare from commercially available VMS, a common coupling agent, and citral, a common terpene, essential oil. This insect repellent/pesticide should provide a cost-effective alternative to synthetic chemical-based systems.

No work had been conducted in search of insecticidal activity of citral and VS-citral. Citral and VS-citral were for the first time evaluated as mosquito repellent and could make excellent acting insecticide, but perhaps their greatest strength would be their use as an insect repellent.

## Figures and Tables

**Table 1:** VS-citral oligomers assigned by MS in the *m*/*z* = 925.2–1590.3 Da.

*m*/*z* (exp.)	Assigned structure (+Na^+^、H^+^、K^+^)	*m*/*z* (pre.)
925.2	T_5_(OH)_3_(OCH_3_)_2_(C_10_H_16_O)_3_H^+^	926.4
941.2	T_5_(OH)_2_(OCH_3_)_3_(C_10_H_16_O)_3_H^+^	940.5
969.2	T_6_(C_10_H_16_O)_3_K^+^	970.5
	T_6_(OH)_2_(C_10_H_16_O)_3_Na^+^	972.5
	T_6_(OH)_4_(C_10_H_16_O)_3_H^+^	968.5
985.2	T_6_(OH)_4_(C_10_H_16_O)_3_Na^+^	990.5
	T_6_(OH)_6_(C_10_H_16_O)_3_H^+^	986.5
1,013.2	[1057.2-C_2_H_4_O]	1,014.7
1,029.2	[1073.2-C_2_H_4_O]	1,032.7
1,057.2	T_7_(OH)(C_10_H_16_O)_3_K^+^	1,058.7
	T_7_(OH)_3_(C_10_H_16_O)_3_Na^+^	1,060.6
	T_7_(OH)_5_(C_10_H_16_O)_3_H^+^	1,056.7
1,073.2	T_7_(OH)_3_(C_10_H_16_O)_3_K^+^	1,076.7
1,101.2	[1145.2-C_2_H_4_O]	1,102.8
1,117.2	[1161.2-C_2_H_4_O]	1,120.9
1,145.2	T_8_(OH)_2_(C_10_H_16_O)_3_K^+^	1,146.8
	T_8_(OH)_4_(C_10_H_16_O)_3_Na^+^	1,148.8
	T_8_(OH)_6_(C_10_H_16_O)_3_H^+^	1,144.8
1,161.2	T_8_(OH)_4_(C_10_H_16_O)_3_K^+^	1164.9
1,189.2	[1233.2-C_2_H_4_O]	1,191.0
1,205.2	[1249.2-C_2_H_4_O]	1,209.0
1,233.2	T_9_(OH)_3_(C_10_H_16_O)_3_K^+^	1,235.0
	T_9_(OH)_5_(C_10_H_16_O)_3_Na^+^	1,236.9
	T_9_(OH)_7_(C_10_H_16_O)_3_H^+^	1,232.9
1,249.2	T_9_(OH)_5_(C_10_H_16_O)_3_K^+^	1,253.0
1,277.2	[1321.2-C_2_H_4_O]	1,279.1
1,321.3	T_10_(OH)_4_(C_10_H_16_O)_3_K^+^	1,323.1
1,365.3	[1409.2-C_2_H_4_O]	1,367.2
1,409.3	T_11_(OH)_5_(C_10_H_16_O)_3_K^+^	1,411.2
1,453.3	[1497.2-C_2_H_4_O]	1,455.4
1,497.3	T_12_(OH)_6_(C_10_H_16_O)_3_K^+^	1,499.4
1,541.3	[1583.2-C_2_H_4_O]	1,543.5
1,590.3	T_13_(OH)_7_(C_10_H_16_O)_3_K^+^	1,587.5

**Table 2:** Repellency of 5% VS-citral against *Aedes albopictus* in laboratory test.

Volunteers	Untreated	Repellency (%) (±SE) at hours after treatment					
	*N*	0 h		1 h		2 h	
		*N*	*R* (%)	*N*	*R* (%)	*N*	*R* (%)
V1	43	0	100	7	83.7	13	69.8
V2	27	0	100	5	81.5	14	48.1
V3	23	1	95.7	11	52.2	14	39.1
V4	68	0	100	13	80.9	16	76.5
V5	38	2	94.7	7	81.6	11	71.1
V6	42	0	100	11	73.8	14	66.7
V7	34	0	100	11	67.6	13	61.8
V8	32	3	90.6	12	62.5	17	46.9
AVG	38.38 ± 2.96	0.75 ± 0.21	97.63 ± 0.72	9.63 ± 0.77	72.98 ± 3.11	12.75 ± 0.71	60 ± 4.23

The number (*N*) of mosquitoes landing on arm of each volunteer was counted per hour for 2 h. Repellency (*R*) was calculated each hour.

**Table 3:** Repellency of 5% citral against *Aedes albopictus* in laboratory test.

Volunteers	Untreated	Repellency (%) (±SE) at hours after treatment					
	*N*	0 h		1 h		2 h	
		*N*	*R* (%)	*N*	*R* (%)	*N*	*R* (%)
V1	23	5	78.3	11	52.2	12	47.8
V2	25	4	84	10	60.0	6	76.0
V3	23	3	87.0	8	65.2	10	56.5
V4	24	0	100	7	70.8	12	50
V5	26	4	84.6	11	57.7	19	26.9
V6	21	0	100	7	66.7	10	52.4
V7	22	0	100	6	72.7	10	54.5
V8	23	4	82.6	10	56.5	13	43.5
AVG	23.38 ± 0.66	2.50 ± 0.71	89.56 ± 3.02	8.75 ± 0.83	62.73 ± 5.31	11.5 ± 1.19	50.95 ± 6.10

The number (*N*) of mosquitoes landing on arm of each volunteer was counted per hour for 2 h. Repellency (*R*) was calculated each hour.

## References

[1] Naters W.V., Carlson J.R. (2006). Insects as chemosensors of humans and crops. Nature.

[2] Trongtokit Y., Rongsriyam Y., Komalamisra N., Apiwathnasorn C. (2005). Comparative repellency of thirty-eight essential oils against mosquito bites. Phytother. Res..

[3] Memon G.Z., Bhanger M.I., Akhtar M. (2008). Adsorption of methyl parathion pesticide from water using watermelon peels as a low cost adsorbent. Chem. Eng. J..

[4] Mitchell D.L., Greinert R., de Gruijl F.R. (1999). Effects of chronic low-dose ultraviolet B radiation on DNA damage and repair in mouse skin. Cancer. Res..

[5] Lu Y., Lou Y., Yen P., Mitchell D., Huang M., Conney A.H. (1999). Time course for early adaptive responses to ultraviolet B light in the epidermis of SKH-1 mice. Cancer. Res..

[6] Kumar P., Mishra S., Malik A., Satya S. (2013). Housefly (*Musca domestica L.*) control potential of *Cymbopogon citratus Stapf.* (*Poales: Poaceae*) essential oil and monoterpenes (citral and 1,8-cineole). Parasitol. Res..

[7] Zhang Q.H., Schneidmiller R.G., Hoover D.R. (2013). Essential oils and their compositions as spatial repellents for pestiferous social wasps. Pest. Manag. Sci..

[8] Giatropoulos A., Papachristos D.P., Kimbaris A., Koliopoulos G., Polissiou M.G., Emmanouel N., Michaelakiset A. (2012). Evaluation of bioefficacy of three Citrus essential oils against the dengue vector *Aedes albopictus* (*Diptera: Culicidae*) in correlation to their components enantiomeric distribution. Parasitol. Res..

[9] Ko K., Juntarajumnong W., Chandrapatya A. (2010). Insecticidal activities of essential oils from fruits of *litsea salicifolia* roxb. ex Wall. Against *sitophilus zeamais* motschulsky and *tribolium castaneum* (Herbst). Pak. J. Zool..

[10] Plarre R., Poschko M., Prozell S., Frank A., Wohlgemuth R., Phillips J.K. (1997). Effects of oil of cloves and citronellol, two commercially available repellents, against the webbing clothes moth *Tineola bisselliella Hum* (*Lepidoptera: Tineidae*). Anz. Schadlingskd. Pfl..

[11] Lin D.R., Hu L.J., You H., Tolbert S.H., Loy D.A. (2014). Comparison of new periodic, mesoporous, hexylene-bridged polysilsesquioxanes with *Pm3n* symmetry versus sol-gel polymerized, hexylene-bridged gels. J. Non-Cryst. Solids.

[12] Lin D.R., Hu L.J., Tolbert S.H., Li Z., Loy D.A. (2015). Controlling nanostructure in periodic mesoporous hexylene-bridged polysilsesquioxanes. J. Non-Cryst. Solids.

[13] Lin D.R., Hu L.J., Li Z., Loy D.A. (2014). Influence of alkylene-bridging group length on mesostructure and porosity in cubic (*Pm3n*) periodic mesoporous bridged polysilsesquioxanes. J. Porous Mater..

[14] Lin D.R., Hu L.J., You H., Williams R.J.J. (2011). Synthesis and characterization of a nanostructured photoluminescent silsesquioxane containing urea and dodecyl groups that can be patterned on carbon films. Eur. Polym. J..

[15] Lin D.R., Zhao Q., Hu L.J., Xing B.S. (2014). Synthesis and characterization of cubic mesoporous bridged polysilsesquioxane for removing organic pollutants from water. Chemosphere.

[16] Hu L.C., Shea K.J. (2011). Organo-silica hybrid functional nanomaterials: How do organic bridging groups and silsesquioxane moieties work hand-in-hand?. Chem. Soc. Rev..

[17] Shea K.J., Moreau J., Loy D.A., Corriu R.J.P., Boury B., Gómez-Romero P., Sanchez C. (2005). Bridged Polysilsesquioxanes. Molecular-engineering nanostructured hybrid organic-inorganic materials. Functional Hybrid Materials.

[18] Loy D.A., Kickelbick G. (2006). Sol-gel processing of hybrid organic-inorganic materials based on polysilsesquioxanes. Hybrid Materials: Synthesis, Characterization, and Applications.

[19] Lin D.R., Hu L.J., Xing B.S., You H., Loy D.A. (2015). Mechanisms of competitive adsorption organic pollutants on hexylene-bridged polysilsesquioxane. Materials.

[20] Hergenrother W.L., Lin C.J., Hilton A.S., Hogan T.E., Brumbaugh D.R. (2010). Reduction of volatile organic compound emission. I. Synthesis and characterization of alkoxy-modified silsesquioxane. J. Appl. Polym. Sci..

[21] Wang H., Lin D., Wang D., Hu L., Huang Y., Liu L., Loy D.A. (2014). Computational and experimental determinations of the UV adsorption of polyvinyl-silsesquioxane-silica and titanium dioxide hybrids. Bio-Med. Mater. Eng..

[22] Wang H., Liu L., Huang Y., Wang D., Hu L., Loy D.A. (2014). Enhancement corrosion resistance of (γ-glycidyloxypropyl)-silsesquioxane-titanium dioxide films and its validation by gas molecule diffusion coefficients using molecular dynamics (MD) simulation. Polymers.

[23] Lichtenhan J.D., Blum F.D., Laine R.M. (2003). Organic/Inorganic Hybrid Materials.

[24] Yang B., Li J., Wang J., Xu H., Guang S., Li C. (2009). Poly(vinyl pyrrolidone-*co*-octavinyl polyhedral oligomeric silsesquioxane) hybrid nanocomposites: Preparation, thermal properties, and *T*_g_ improvement mechanism. J. Appl. Polym. Sci..

[25] Bai X., Zhang C., Liu B., Jiang C., Mu J. (2012). Synthesis and thermal stability of hybrid polymers using UV photopolymerization based on polyhedral oligomeric silsesquioxanes. High Perform. Polym..

[26] Seno M., Hasegawa M., Hirano T., Sato T. (2005). Radical polymerization behavior of trimethoxy- vinylsilane. J. Polym. Sci. Pol. Chem..

[27] Rzaev Z.M., Bryksina L.V., Kyazimov S.K., Sadykh-Zade S.I. (1972). Radical copolymerization of maleic anhydride, styrene, and vinyltriethoxysilane. Vysokomol. Soedin..

[28] Zhang X.W., Hu L.J., Sun D.Z., Zhao W. (2008). Study of three-dimensional configurations of organic/inorganic hybrid nanostructural blocks: A quantum chemical investigation for cage structure of (γ-glycidoxypropyl)-silsesquioxanes. J. Mol. Struct..

[29] Chen X.D., Wang D., Lin D.R., Zhang Q., Hu L.J. (2010). Transparency of modified vinyl-silsesquioxane films and its validation by computing energy-band structure. J. Mater. Res..

[30] Logan J.G., Stanczyk N.M., Hassanali A., Kemei J., Santana A.E.G., Ribeiro K.A.L., Pickett J.A., Mordue A.J. (2010). Arm-in-cage testing of natural human-derived mosquito repellents. Malar. J..

[31] Bernstein I.L., Li J.T., Bernstein D.I., Hamilton R., Spector S.L., Tan R., Sicherer S., Golden D.B.K., Khan D.A., Nicklas R.A. (2008). Allergy diagnostic testing: an updated practice parameter. Ann. Allerg. Asthma. Im..

[32] World Health Organization Pesticide Evaluation Scheme (WHOPES) (2006). Guidelines for Efficacy Testing of Mosquito Repellents for Human Skin.

[33] Schreck C.E. (1977). Techniques for the evaluation of insect repellents: a critical review. Annu. Rev. Entomol..

[34] Biondi M., Borzacchiello A., Netti P.A. (2010). Isothermal and non-isothermal polymerization of methyl methacrylate in presence of multiple initiators. Chem. Eng. J..

[35] Wang B., Ramirez A.P., Slade J.J., Morken J.P. (2010). Enantioselective synthesis of (−)-sclerophytin A by a stereoconvergent epoxide hydrolysis. J. Am. Chem. Soc..

[36] Ueno T., Masuda H., Ho C.T. (2004). Formation mechanism of *p*-methylacetophenone from citral via a tert-alkoxy radical intermediate. J. Agric. Food. Chem..

[37] Vanbrugg E. (1968). Free radical additions 2. Free radical cyclization of citronellol. Recl. Trav. Chim. Pay-B..

[38] Erba I.E., Fasce D.P., Williams R.J.J., Erra-Balsells R., Fukuyama Y., Nonami H. (2003). Poly-(silsesquioxanes) derived from the hydrolytic condensation of organotrialkoxysilanes containing hydroxyl groups. J. Organomet. Chem..

[39] Zapata A., Oller I., Sirtori C. (2010). Decontamination of industrial wastewater containing pesticides by combining large-scale homogeneous solar photocatalysis and biological treatment. Chem. Eng. J..

[40] Fasce D.P., Williams R.J.J., Erra-Balsells R., Ishikawa Y., Nonami H. (2001). One-Step synthesis of polyhedral silsesquioxanes bearing bulky substituents: UV-MALDI-TOF and ESI-TOF mass spectrometry characterization of reaction products. Macromolecules.

[41] Nibbering N.M. (2004). The McLafferty rearrangement: A personal recollection. J. Am. Soc. Mass Spectrom..

[42] Gross M.L. (2004). Focus in honor of Fred McLafferty, 2003 Distinguished Contribution awardee, for the discovery of the "McLafferty Rearrangement”. J. Am. Soc. Mass Spectrom..

